# Correspondence

**Published:** 1904-07-15

**Authors:** Lee S. Smith

**Affiliations:** Secretary American Dental Trade Association


### CORRESPONDENCE.

Editor of the Dental Register:

I enclose copy of a resolution introduced and unanimously adopted by the members of the American Dental Trade Association held in St. Louis last week, a copy of which I would request should appear in the next issue of your journal.

Whereas, In the estimation of our Association, which is composed of the representative manufacturers of and dealers in dental supplies in the United States, the amendment to patent laws introduced in Senate bill 4256, House bill 6771, will remove a class of patents which have long caused annoyance to the dental profession; therefore be it

*Resolved,* That the American Dental Trade Association heartily endorse the amendment and urge favorable action thereon, and that the secretary be instructed to notify the committee having the amendment in consideration of such action.

Lee S. Smith,
*Secretary American Dental Trade Association.*

